# Computational Chemistry for the Identification of Lead Compounds for Radiotracer Development

**DOI:** 10.3390/ph16020317

**Published:** 2023-02-18

**Authors:** Chia-Ju Hsieh, Sam Giannakoulias, E. James Petersson, Robert H. Mach

**Affiliations:** 1 Division of Nuclear Medicine and Clinical Molecular Imaging, Department of Radiology, Perelman School of Medicine, University of Pennsylvania, Philadelphia, PA 19104, USA; 2 Department of Chemistry, University of Pennsylvania, Philadelphia, PA 19104, USA

**Keywords:** radiotracer, radiopharmaceutical, computer-aided drug design, CADD, virtual screening, in silico, docking, molecular dynamic simulations, pharmacophore, QSAR, ADMET, positron emission tomography, PET, BOILED-Egg plot

## Abstract

The use of computer-aided drug design (CADD) for the identification of lead compounds in radiotracer development is steadily increasing. Traditional CADD methods, such as structure-based and ligand-based virtual screening and optimization, have been successfully utilized in many drug discovery programs and are highlighted throughout this review. First, we discuss the use of virtual screening for hit identification at the beginning of drug discovery programs. This is followed by an analysis of how the hits derived from virtual screening can be filtered and culled to highly probable candidates to test in in vitro assays. We then illustrate how CADD can be used to optimize the potency of experimentally validated hit compounds from virtual screening for use in positron emission tomography (PET). Finally, we conclude with a survey of the newest techniques in CADD employing machine learning (ML).

## 1. Introduction

Radiotracers require high affinity for the target of interest, low off-target binding, and suitable pharmacokinetic properties to be useful in in vivo imaging studies. The design of new radiotracers that meet these criteria is incredibly difficult, time consuming, and costly. However, computer-aided drug design (CADD) approaches, including virtual screening (VS) and ligand optimization, have been utilized to increase the efficiency of identifying and optimizing novel compounds into lead radiotracers [1,2,3,4].

CADD is often categorized into two major areas, structure-based and ligand-based approaches [5]. Structure-based approaches, sometimes called “physics-based approaches”, require a 3-dimensional (3-D) structure of the macromolecular target of interest as an input. These methods are achieved through a docking procedure and rely on a force field and an empirical-based score function to determine the fitness and putative binding poses of a ligand at a particular site on a target of interest. On the other hand, ligand-based approaches in CADD are focused on learning relationships between properties in candidate molecules and a particular experimental value of interest, such as binding affinity. Ligand-based approaches can be used when there is not an available 3-D structure of the target of interest and are particularly useful when there is a set of compounds that show at least modest affinity.

Both structure and ligand-based approaches are useful at the level of VS and subsequent optimization. Additionally, in situations where 3-D structures of the target of interest are available, there has been demonstrated value in combining the outputs of these two different approaches [6]. In the last decade, both structure- and ligand-based methods have been improved with the use of machine learning (ML) [7,8,9,10,11]. This review will provide a brief introduction to these methods, including examples where they have been successfully utilized in radiopharmaceutical development to date.

## 2. Virtual Screening

### 2.1. Virtual Screening Overview

**Figure 1:**
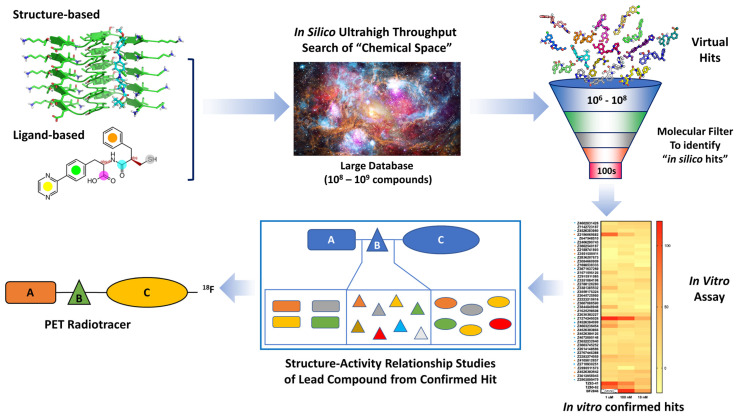
Workflow from VS to lead compounds identification for radiotracer development. “A, B, and C” in the last two steps of the workflow are represented as the fragment “A”, “B”, and “C” for structure–activity relationship studies.

VS is the process of performing the computational equivalent of the experiments of a high-throughput screen to narrow the pool of candidate ligands before doing subsequent experiments in the laboratory [12,13]. When a new drug discovery program is started without any or with only very limited preliminary data, it is typically not possible to rationally design ligands. Therefore, VS is typically used as the first CADD method in a pipeline as it can rapidly test a large number of compounds computationally, reducing time and cost by limiting the number of compounds that must be synthesized or purchased. VS is usually performed using structure-based methods but can also be performed via ligand-based methods if there is at least one known hit [14]. Most often, VS is performed at an ultra-high-throughput scale (millions to billions of compounds) employing previously enumerated and purchasable chemical libraries or in-house VS libraries [15]. A standard VS workflow for the development of new radiotracers is described in Figure 1.

To date, in radiopharmaceutical development, VS approaches have been performed mainly on G protein-coupled receptors, protein kinases and insoluble protein aggregates such as alpha-synuclein and microtubule-associated protein tau. The details of each approach and their application in radiotracer development is discussed below. Table 1 summarizes the past two decades worth of virtual screens that have identified small molecules having a high affinity against their target of interest.

### 2.2. Structure-Based Virtual Screening

Protein structures for structure-based VS are typically obtained from the Protein Data Bank (PDB) [48]. The experimental structures in the PDB are mainly derived from X-ray crystallography, cryo-electron microscopy (cryo-EM), or nuclear magnetic resonance spectroscopy (NMR). In situations where the PDB does not contain a structure of the target of interest, there are still reasonable avenues to structure-based VS. Homology modeling is the process of computationally generating a model of a 3-D structure from amino acid sequence alone [49]. Historically, homology modeling was limited to proteins with high sequence similarity to other proteins with an already solved 3-D structure. This homology modeling was most often performed using programs such as the Rosetta Modeling Suite [50] or web servers such as SwissProt [51]. In 2021, homology modeling took a major step forward with the release of AlphaFold 2 [52], an ML-based approach, which demonstrated a remarkable ability to predict 3-D structures from sequence alone in the 14th Critical Assessment of Structure Prediction (CASP) competition. Computational chemists now have the luxury of working with AlphaFold structures for almost any protein target in the human genome (these models can easily be found in Uniprot [53]). AlphaFold structures are particularly useful as they include a representation of model confidence across the structure allowing the computational chemist to know whether they can confidently utilize the model for VS.

A protein structure alone is not sufficient to begin VS. A potential binding pocket for the ligands must also be identified. In many cases, the radioligand binding site may be the same as the binding site of an enzyme substrate or receptor ligand, the orthosteric binding site (OBS). These are easily identified based on prior literature for the target protein, and structures with a small molecule bound may be available on the PDB. In other cases, a secondary binding site (SBS) may be more advantageous for radioligand binding, and other methods, such as photoaffinity labeling, may be useful in identifying such SBSs. The most challenging cases are protein targets that are not enzymes or receptors that have no intrinsic small molecule binding activity. Amyloid-type fibrils of proteins such as alpha-synuclein and tau are representative of this most challenging category, but these too can be made tractable by the identification of potential binding sites through combinations of computational docking or binding site prediction programs, such as MOLE [54], DoGSiteScorer [55], SiteFiNDER [56], and DrugPred [57], along with photoaffinity labeling.

With a 3-D structure of the protein of interest and a desired binding pocket identified, structure-based VS can be performed after selecting which chemical library to screen and which procedure to utilize. Computational chemists have access to a variety of ultra-large libraries including ZINC [58,59,60] and ChEMBL [61], and other compound databases from vendors, such as Enamine Ltd. [62,63], WuXi AppTec [64], ChemDiv Inc. [65], Asinex Corp. [66], ChemBridge Corp. [67], and Mcule Inc. [68]. Each library covers its own unique chemical space, but the largest library is from Enamine and extends beyond 31 billion compounds. With respect to procedures, the computational chemist also has access to a variety of methods including docking and pharmacophore modeling.

Among docking procedures, AutoDock [69,70], AutoDock Vina [71], Glide [72], DOCK [73], GOLD [74], FRED [75], and RosettaLigand [76] represent a subset of commonly used programs. No matter the procedure, docking is used to predict the orientation and conformation of a small molecule as it interacts with a protein based on a fitness criterion. Each docking procedure seeks to maximize a different metric. For instance, AutoDock Vina uses a scoring function made up five terms that encompass physical properties such as steric, hydrophobicity, and hydrogen bonding [71]. A typical docking-based VS will screen an entire library and only continue to investigate roughly the top 0.1% of fitness scores based on the score function of the docking program used [12].

**Figure 2:**
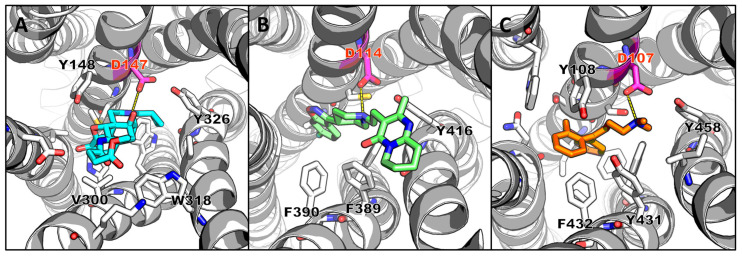
Illustration of the key interactions between amino acid residues in the binding site and the crystallographic ligand. (**A**) µ-opioid receptor and a morphinan antagonist (PDB ID: 4DKL) [77], (**B**) dopamine D2 receptor and risperidone (PDB ID: 6CM4) [78], and (**C**) histamine H1 receptor and doxepin (PDB ID: 3RZE) [79].

Although the exact binding mode of active ligands to the target protein is not required for docking studies, prior knowledge of the key interactions between amino acid residues in the binding site and known active ligands is useful. This information can be used to exclude unfavorable molecules in the virtual screen. Examples where this strategy has been successful include the polar interactions between small molecules and Asp147 of the µ-opioid receptor [16] (Figure 2A), Asp114 of dopamine D2 receptors [27] (Figure 2B), Asp107 of the histamine H1 receptor [18] (Figure 2C), Asp94 of the histamine H4 receptor [19], Glu164 and Asp184 of Mas-related G protein-coupled receptor X2 [17], and His324 of choline acetyltransferase [28]. The hit rate of docking-based VS is between 5% and 20%, and it can be as high as 80% since, in general, the binding affinity cut-off of hit compounds is usually set in the micromolar range (Table 1). Only a few of the studies were able to identify hit compounds having affinities in the nanomolar range [18,19,20,27,30].

Although the potency of compounds from docking-based VS is not ideal to serve as radiotracers per se, these can be obtained via additional structure–activity relationship (SAR) studies. The SAR studies can be performed in silico by screening structural analogs of the best hits from the high-throughput screen, or from traditional organic synthesis (Figure 1). This generally requires an improvement in binding affinity of 10-fold or higher from the initial in silico hit as the binding affinity aims at 1–10 nM or better to serve as a radiotracer [16,17,22]. For example, Manglik et al. optimized the best hit from the virtual screen by using a combination of ordering additional commercially available analogs of the hit compound and organic synthesis to improve the binding affinity for µ-opioid receptors from 2.5 µM to 1.1 nM [16]. Another example is the study by Weiss et al. who utilized a docking campaign aimed at identifying selective compounds for dopamine D2 receptors versus serotonin 5-HT_2A_ receptors, and κ-opioid receptors versus µ-opioid receptors. The approach was able to identify compounds having a high affinity for all four receptors, but it did not lead to D2 or κ-opioid selective ligands [80]. The authors concluded that docking studies can be used to identify ligands having a high affinity for a target protein, but this is not the best method for improving selectivity for a small molecule that binds to multiple protein targets. The failure of selectivity prediction is due to the simple scoring function of docking that could only provide the relative activities in a series of ligands for the same protein target, but not accurate enough to predict absolute binding energies or affinities in comparison with different protein targets [81].

Docking, while effective, is very computationally intensive, since the candidate ligand and the protein, or at least the binding site residues, must be represented. While rigid body docking is more efficient, it can misrepresent the binding interaction if the conformers of the ligand or the protein sidechains are not correct, and sampling multiple conformations further increases computational expense [82]. For that reason, structure-based 3-D pharmacophore models are often applied upstream of docking as they can be computed more quickly [83]. Pharmacophore models seek to identify hits by comparing the structural features of reference compounds (known active compounds) with database molecules in a compound library. If there are available co-crystal structures, database screening will be used against the active conformation directly. Without this information, the pharmacophore method uses docking of the reference compounds to obtain proposed active conformations and build a 3-D pharmacophore model for VS [32,33,34,35,36,39]. With the pharmacophore model in place, VS can be very quickly achieved as database molecules are only compared to the reference without the need for physics-based docking to the protein. Conformers of the screened molecules are overlayed with the 3-D pharmacophoric model of the reference compounds. One important consideration in this method is that database molecules may align well but may still exhibit structural features that are unfavorable in the protein binding site (e.g., adverse steric interactions with key amino acid residues). Therefore, studies using the 3-D pharmacophore-based method for VS are often followed or combined with traditional docking studies to filter out compounds exhibiting unfavorable interactions with the protein [35,36,37,39].

**Figure 3:**
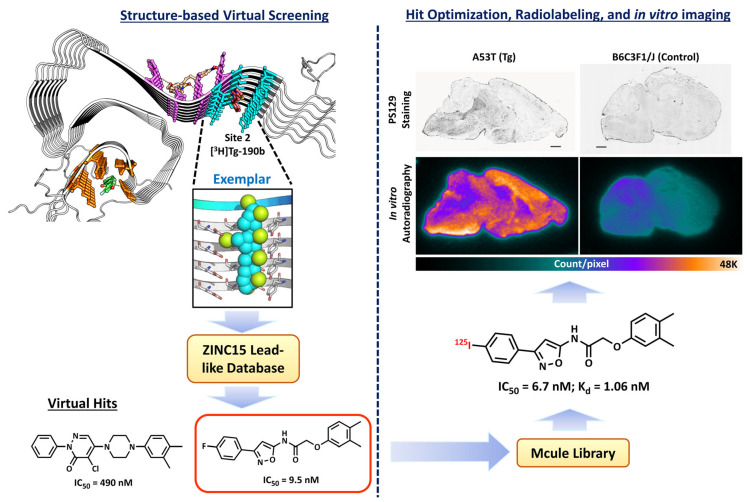
A summary workflow from Ferrie et al. that identified lead compounds from structural-based VS.

A similar but distinct pharmacophore strategy to the 3-D pharmacophore models described above is Gaussian sphere alignment to pseudomolecules [2,84,85]. This procedure is used when co-crystal structure information is not available and involves the generation of a pseudoligand that fills the volume of the putative binding site and has complementary chemical properties [86]. In this procedure, database molecules are aligned to the pseudoligand, and fitness is determined by how much the electron density of a database molecule overlaps with the pseudoligand, as well as the specific overlap of similar heteroatoms. Ferrie et al. [2] used this method to generate pseudoligands (termed “exemplars”) for Sites 2 and 9 of alpha-synuclein fibrils, which were putative binding sites for radioligands known to bind with high affinity to this target [1]. Two virtual hits having binding affinities (IC_50_-values) of 10 and 490 nM for alpha-synuclein fibrils on Site 2 were successfully identified using this method. A ligand-based similarity search was then conducted on the best virtual hits, and this effort identified a lead compound that had an IC_50_ of 3 nM in displacing the [^3^H]tg-190b Site 2 screening ligand. The lead compound was further radiolabeled with ^125^I for in vitro autoradiography and displayed high specific binding to alpha-synuclein pathology in A53T alpha-synuclein transgenic mouse brain and low binding in the control mouse brain (Figure 3). This is the only published report using the pseudoligand method in radiotracer development.

In its more general application, the hit rate of the structure-based 3-D pharmacophore approach and the pseudoligand method is between 0.7% and 46% when a “hit” is identified as having a binding affinity in the sub-micromolar to micromolar range (Table 1). As with docking-based VS, additional SAR studies on the virtual hits are needed to improve the potency of the compounds to the nM range, which is required for serving as lead compounds for radiotracer development [2,34,35].

Finally, although not yet used for radiotracer development, recent ML-based advancements in VS are potentially promising. Adeshina et al. demonstrated that ML can reduce the false positive rate of VS by employing a structure-based ML model called vScreenML [40]. This model utilizes features from Rosetta [50], SZYBKI (OpenEye Scientific Software), ChemAxon [87], BINANA [88], and RF score [8] that combines the chemical properties of ligands and protein–ligand interactions to predict whether a protein–ligand complex is a real crystal structure or if it is a decoy. This method was able to identify a virtual hit having binding affinity of 173 nM for acetylcholinesterase, and the hit rate of the study is 43%. vScreenML may be a useful tool for future radiotracer development when VS needs to be performed.

### 2.3. Ligand-Based Virtual Screening

When a target protein structure is not known (and AlphaFold models are uncertain), or the location of the binding site in the target protein is not known, ligand-based VS techniques can be applied. The only requirement of ligand-based VS is the structure of at least one reference compound for the protein of interest. The most common ligand-based VS methods are chemical fingerprinting and quantitative structure–activity relationships (QSAR) [89].

**Figure 4:**
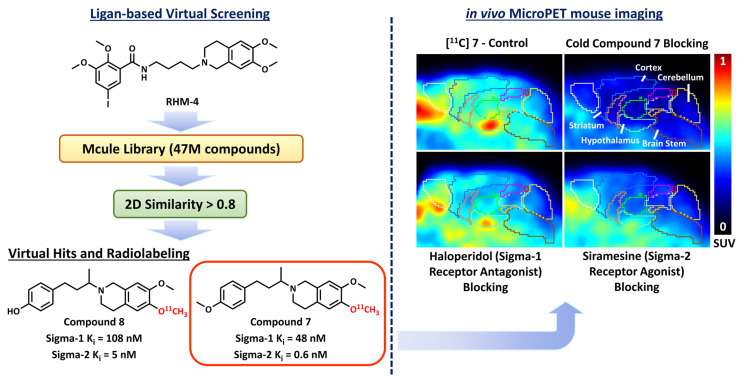
A summary workflow from Kim et al. that identified lead compounds from ligand-based VS.

Chemical fingerprinting methods identify possible hits by conducting a structural similarity score, which is calculated by comparing the 2-D and/or 3-D “fingerprints” of the screening compounds to those present in the reference compound(s) [90,91]. Two-dimensional fingerprints are vector representations of molecules and come in different varieties but are ultimately based on substructures. Morgan fingerprints are the most commonly used, but Daylight, MACCS, and Topological fingerprints are also commonly used 2-D representations that are easily computable with the Python library RDKIT [91]. Vector representations of molecules allow for easily computable quantitative measures of similarity. The most commonly used metric is called the Tanimoto similarity, which is a quantity bounded between 0 and 1 [92,93]. Three-dimensional fingerprints differ from 2-D fingerprints only in that the substructure search procedure is not limited to the 2-D representation of the molecule but uses a shell radius on a low-energy 3-D conformer of the molecule to produce the vector representation. Kim et al. used a selective compound, RHM-4, for sigma-2 receptors as the reference compound for 2-D fingerprint similarity screening and identified multiple virtual hits that had binding affinities in nanomolar range for the sigma-2 receptor [3] (Figure 4). The top two virtual hits had sub-nanomolar binding affinities for the sigma-2 receptor and 20- to 80-fold selectivity over sigma-1 receptors. The two lead compounds were radiolabeled with carbon-11, and in vivo microPET imaging studies demonstrated high specific binding to sigma-2 receptors in a mouse brain (Figure 4).

QSAR modeling is a method that evaluates the correlation between the structural properties of known compounds and their biological activities. The properties of the molecules that go into making the QSAR model N-dimensional are typically computed or annotated from a literature database. QSAR can then be incorporated into VS by selecting database molecules that show high predicted binding affinity based on the QSAR model. Floresta et al. conducted a combination of 2-D and 3-D QSAR methods in VS and successfully identified virtual hits having binding affinities in the sub-nanomolar range for sigma-2 receptors [44].

Ligand-based VS is commonly used in the second round of VS; in this case, the lead compounds that were identified from the initial screen are used as the reference compounds. A structural similarity search is then conducted to identify new hits with similar structures, but it is also possible to identify new scaffolds using this method [2,22]. In general, the hit rate of the ligand-based VS method is 1.9% to 33% when the binding affinity threshold for hit compounds is set in the sub-micromolar to micromolar range (Table 1).

## 3. Biological Property Prediction and Hit Filtering

The ever-growing size of chemical libraries poses practical challenges for CADD and for the medicinal chemist left to work with the VS data. Manual inspection of screens on ultra-large libraries has become intractable (potentially millions of “hits”); therefore, it is very common to apply filters on chemical properties or predictors of biological availability to narrow down the size of libraries prior to screening or initial hits following the screening.

There are a number of methods one can use to select compounds from the initial virtual screen to create a smaller library for high-throughput screening. An early method when libraries were smaller was to simply perform an intensive visual inspection to identify compounds of interest [2,3,16,17,18,19,20,24,27,28,32]. However, for visual inspection to be effective, it typically requires specific domain expertise and is prone to certain biases when selecting compounds. To avoid bias from manual selection, methods such as culling based on docking scores or structural similarity can be used to select top-ranking compounds obtained from the virtual screen prior to submission for in vitro binding affinity measurements [29,30,41,42,44]. Another technique typically applied after culling based on a docking or similarity score would be compound clustering. Clustering is used to bin very similar compounds together so that by selecting representative members of each cluster only, the maximum number of highly diverse and informative experiments can be performed with a small number of purchased or synthesized compounds [94].

In addition to ranking based on docking or similarity scores and clustering, many unwanted hits can be filtered out using chemical property and biological activity filters. These can be calculated prior to conducting the virtual screen. Applying these filters to the compound libraries prior to VS will reduce computing time by narrowing the library to molecules more likely to have bioactivity [32,42]. When used after VS, it can represent a key step in narrowing down the number of compounds for experimental validation [46,47]. Compound libraries such as ZINC15 [60], ChEMBL [61], and Enamine REAL database [62] provide basic chemical properties and various biological indicators for each molecule in their database.

Among chemical property-based filters, Lipinski’s rule of five [95] is the most common method for selecting compounds with drug-like properties. Compounds having a molecular weight lower than 500, logP lower than 5, less than 5 hydrogen-bond donors, and less than 10 hydrogen-bond acceptors are predicted as having drug-like properties. Additional rules based on the number of rotatable bonds, total polar surface area, lowest pKa, and solubility in water have been used to exclude potentially inactive compounds.

Similar to chemical property filters, biological behavior predictions can be used to filter unwanted molecules from libraries. Biological behaviors are typically a mixture of general rules and parameters of specific importance on a per-program basis. For instance, molecules that act as pan-assay interference compounds (PAINS) would ideally be filtered before VS [96]. Other general characteristics typically computed are absorption, distribution, metabolism, excretion, and toxicity (ADMET) values [97]. Each of these categories is represented with various specific metrics, such as Caco-2 membrane permeability or LD_50_. Prediction of these values for compounds in libraries can help to identify hits with strong physiochemical profiles. In the field of radiotracer development for neuroimaging, ADMET predictions have been applied to predict blood-brain barrier (BBB) permeability. Steen et al. have reviewed multiple in silico approaches for predicting BBB permeability in the application of central nervous system (CNS) radiotracers [98]. The predictive accuracy of the scoring systems from multiparameter optimization (MPO), including CNS-MPO [99] and CNS PET MPO [100], has been evaluated in a set of radiotracers, and its predictive accuracy is approximately 60 to 70% with 66 to 75% sensitivity (true positive rate) and 30 to 45% specificity (true negative rate) [98].

The brain or intestinal estimated permeation method (BOILED-Egg) provides a graphical model for BBB permeability by simply plotting the lipophilicity (WLogP) and topological polar surface area (tPSA) of compounds in a scatter plot [101]. Compounds located in the “yolk” of the BOILED-Egg plot are predicted to cross the BBB, and those located in the “egg white” are predicted to have gastrointestinal absorption. While the BOILED-Egg plot represents a simple and intuitive method for predicting BBB penetration, there are other ML-based approaches that have shown a strong ability to predict BBB penetration at the cost of interpretability. Kumar et al. demonstrated that a transfer learning approach employing a deep neural network, DeePred-BBB, was able to achieve 98% accuracy on a dataset of over 3000 tested compounds [102].

**Figure 5:**
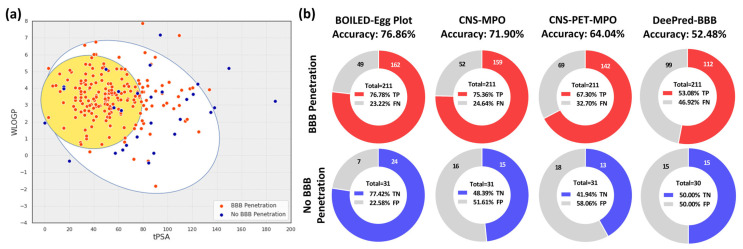
(**a**) BOILED-Egg plot of the testing radiotracer dataset, including 211 BBB-penetrated and 31 not BBB-penetrated radioligands from the literature. (**b**) Pie charts of true positive (TP), false negative (FN), true negative (TN), and false positive (FP) rates for BOILED-Egg plot, CNS-MPO, CNS PET MPO, and DeePred-BBB. The total number of not BBB-penetrated compounds for DeePred-BBB is 30 due to the conversion failure of one of the compounds from the program.

Below, we have expanded the radiotracer library from the collection of Steen et al. by adding additional radiotracers from the literature (Table S1) to test the predictive accuracy of BBB penetration of the BOILED-Egg plot and DeePred-BBB. The WLogP and tPSA for the BOILED-Egg plot were calculated by using the SwissADME web server [103], and the predictions of DeePred-BBB were computed by using the model that has been provided at GitHub by Kumar et al. [102]. The SMILES strings of radiotracers and their related calculations that have been used to evaluate each BBB permeability prediction method are listed in the Supplemental Data (Table S1). The BOILED-Egg plot of the test radiotracer dataset is shown in Figure 5a. The predictive accuracy of BBB permeability for known radiopharmaceuticals is 76.9%, with 76.8% sensitivity and 77.4% specificity (Figure 5b). The CNS-MPO and CNS PET MPO scores were also calculated for the same dataset. The predictive accuracy for CNS-MPO is 71.9% with 75.4% sensitivity and 48.4% specificity; the predictive accuracy for CNS PET MPO is 64.0% with 67.3% sensitivity and 41.9% specificity (Figure 5b). The predictive accuracy for DeePred-BBB is 52.5% with 53.1% sensitivity and 50.0% specificity (Figure 5b), significantly worse than the reported predictive accuracy. This indicates that the DeePred-BBB may be improved for use in radiotracer development with retraining on the radioligand dataset.

It should be noted that the biological activity predictions do not predict binding affinity for the target protein. When used following the process of VS to filter compounds, this approach may provide guidance in the design of new structures and SAR studies by predicting their biological properties from the CNS-MPO score or their location in the BOILED-Egg plot [101,104].

## 4. Hit Compound Optimization

### 4.1. Structure-Based Hit Compound Optimization

CADD methods for structure-based hit optimization include docking [82], molecular dynamics simulations (MDS) [105], and physics-based ML models [106]. Oftentimes, MDS is performed on an initial starting structure produced from a faster docking procedure since MDS is very computationally intense. MDS is a technique that simulates the dynamic interactions between a small molecule and a binding site within a protein. Both molecular docking and MDS studies have been used to identify the important interactions between a small molecule and key amino acid residues in a protein that contribute to the high-affinity binding of the ligand for the protein. These methods also reveal the active conformations of the ligands that are important in the binding of flexible ligands to the target protein [104,107,108,109,110]. This approach has been utilized in the development of ligands for G protein-coupled receptors, such as the dopamine D2 and D3 receptors, by investigating the ligand binding profiles in two different binding sites in the receptor, the OBS and SBS. Optimization of the binding properties of ligand fragments interacting with the OBS and SBS led to the generation of “bitopic ligands” having a high affinity and selectivity for the D3 receptor [111,112,113,114,115,116].

While MDS provides valuable representations of protein and ligand dynamics, accurate representation of solvent interactions, which can drive binding, remains a challenge. It has been shown that the binding free energy values produced from MDS can be made more accurate using the molecular mechanics Poisson–Boltzmann surface area (MM/PBSA) or molecular mechanics generalized Born surface area (MM/GBSA) methods [117,118]. These methods have been utilized to characterize the binding profiles, potentially multiple binding sites, multitargets, and the off-target binding of high potency ligands for G protein-coupled receptors [30,109,113,119,120], kinase [108] and insoluble protein aggregates [121,122,123,124,125].

Recently, ML-based advancements in structure-based compound optimization have been developed for the accurate prediction of absolute protein-ligand binding affinity. Brown et al. benchmarked BCL-AffinityNet, a graph neural network-based deep learning model on the CASF and PDBBind datasets showing best or very close to best predictive powers [106]. Going forward, BCL-AffinityNet could be used to help guide SAR and hit optimization in radiotracer development.

**Figure 6:**
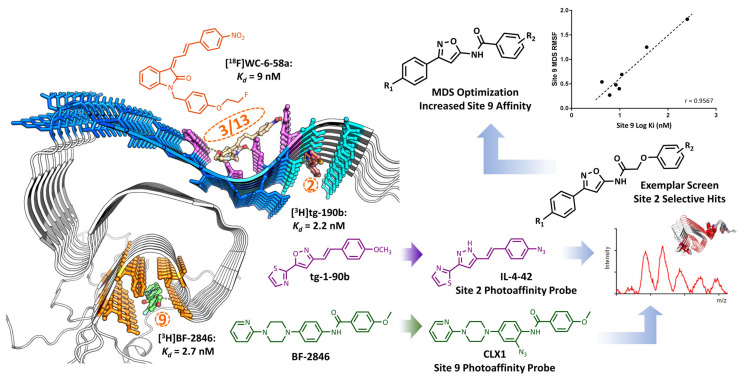
Three putative alpha-synuclein binding sites, Sites 2, 3/13, and 9, identified from the blind docking studies. Site 2 and Site 9 were confirmed via in vitro photo-cross-linking and mass spectrometry studies. [^3^H]tg-190b and IL-4-42 are the radioligand and photoaffinity probes for Site 2. [^3^H]BF-2846 and CLX1 are the radioligand and photoaffinity probes for Site 9. Site 2 and Site 9 probes were used to test in silico hits from the Exemplar screen and Site 9 optimization based on MDS.

The docking followed by the MDS method has been used recently in the development of radiotracers targeting insoluble protein aggregates, including alpha-synuclein and tau. This method initially used blind docking studies to reveal putative binding sites in the protein based on the fibrillar structures of alpha-synuclein and tau [1,121,123,124]. Using the available solid-state NMR structure of alpha-synuclein, Hsieh et al. conducted docking and MDS studies to identify three putative binding sites in alpha-synuclein for radioligands used in vitro binding assays for screening small molecules capable of binding to this protein (Figure 6). The location of the putative binding sites 2 and 9 was confirmed via in vitro crosslinking and mass spectrometry studies using photoaffinity probes based on the different radioligands, [^3^H]tg-190b and [^3^H]BF-2846 [1] (Figure 6).

The locations of these binding sites were used in Ferrie et al. for VS using the “Exemplar” method to identify new, higher affinity lead compounds, as described above [2]. The site selectivity of these VS-derived compounds was then confirmed through photo-crosslinking, showing that the Exemplar pseudoligands indeed faithfully represented the binding site interactions [2,126]. Notably, the Site 2 compounds identified in the VS represent scaffold hops that are chemically distinct from tg-190b, with moderate 2-D similarity (Tanimoto score of MACCS fingerprints: 0.48–0.55) (Figure 6). Subsequent MDS studies have further improved the affinity and Site 9 selectivity of the hits from VS [127]. Site 9 affinity from MDS was computed from the root mean squared fluctuation (RMSF) of compounds docked to Site 9 to determine the stability of the ligand in the binding pocket. Then, the RMSF values were further compared with experimental binding affinity to establish a correlation that could be used to successfully predict new compounds with increased Site 9 affinity (Figure 6). Compounds from Site 9 show greater promise as Parkinson’s disease PET imaging leads, where Site 2 availability may be compromised by post-translational modifications [128,129]. Thus, the ability to tune binding affinity for a specific site through CADD is extremely valuable in radiotracer development for Parkinson’s disease and related synucleinopathies. More generally, these studies illustrate how multiple methods can be used to iteratively improve the affinity and selectivity.

In radiotracer development for the tauopathies, blind docking and MDS studies were performed on radioligands that have been used in translational imaging studies to obtain insight to explain the confusing behavior of tau ligands in different radioligand binding assays. As in the case of alpha-synuclein, these studies identified multiple putative binding sites for radiotracers within the tau fibril structure [123,124]. This approach was also used to investigate the binding profile for radiotracers to the different tauopathies, such as Alzheimer’s disease, corticobasal degeneration, progressive supranuclear palsy, chronic traumatic encephalopathy, and Pick’s disease [121,122]. A more comprehensive understanding of the precise location of the ligand binding sites in the different tau structures will be necessary for the design of high affinity and selective radioligands specific to the different tauopathies. Given the availability of numerous patient-derived tau fibril structures from cryo-EM, the iterative approach described above for alpha-synuclein could likely be applied to tau as well [130,131,132,133,134].

### 4.2. Ligand-Based Hit Compound Optimization

Similar to VS, QSAR studies are a valuable tool for ligand-based hit compound optimization. This approach has been employed in radiotracer development for multiple targets, including dopamine receptors [135,136,137,138,139,140], serotonin receptors [141], sigma receptors [142,143], beta-amyloid fibrils [4,144,145], and cancer-related kinases or receptors [146,147,148,149]. A QSAR model that is built to investigate ligand fragments that contribute to the binding affinities for multiple proteins, such as target and off-target proteins, can be used to predict the binding affinity for a protein of interest as well as selectivity versus off-target binding. Using information acquired from a QSAR model, it was possible to design new ligands for dopamine D3 receptors having a high affinity and selectivity over dopamine D2 receptors [135] or other off-target proteins such as endocannabinoid receptors [138]. Yang et al. used QSAR models to successfully predict the binding affinities of two ligands for beta-amyloid plaques; they then radiolabeled the compounds with ^18^F for PET and ^125^I for single-photon emission computed tomography (SPECT) in small animal imaging studies [4].

## 5. Limitations and Conclusions

CADD approaches provide insight into protein–ligand binding interactions as well as relevant chemical properties to guide the identification and further development of high-affinity radioligands. VS is a highly effective tool for the identification of novel active chemical matter at the beginning of drug discovery programs, or as the alpha-synuclein studies illustrate, to scaffold hop from existing hits. However, it is not without its limitations. In particular, structure-based virtual screens require an input 3-D protein structure and access to extensive computing time and power. Additionally, docking typically does not take protein dynamics or implicit solvation into account, and hit rates can be quite variable depending on the suitability of the score functions for a particular target. Each VS procedure is different and requires specific knowledge of the desired ligand properties and binding modes for the evaluation of hits, as well as the computational methods and hardware requirements. While small-scale docking campaigns (hundreds to thousands of compounds) can be run on an advanced desktop computer, the existence of million-to-billion-member compound libraries prompts the use of ultra-high-throughput methods. For these, institutional clusters or commercial cloud-based computing resources are required, and expertise in choosing the appropriate system should be sought. For example, a large CPU/GPU cluster will be highly effective for docking but will be tremendously slower than a GPU cluster for running Gaussian overlap computes. Database and hit filtering from VS are critical steps in a drug discovery pipeline that can significantly reduce time, cost, and effort when applied effectively. The use of chemical property filters and biological predictors is highly effective at improving the hit rate of virtual screens and can remove bias that emerges from visual inspection. Since hits from VS typically demonstrate potencies in the micromolar to the mid-nanomolar range, additional compound optimization is required for radiopharmaceutical development. Structure-based compound optimization with docking, MDS, and BCL-AffinityNet alongside ligand-based QSAR models have proven effective in many drug discovery programs. Taken together, the studies in this review employing the ever-expanding computational chemistry toolkit represent a bright future for radiotracer development in the years to come.

## Figures and Tables

**Table 1:** Summary of the VS in the identification of small molecules for different protein targets.

Method	Target	# of Compounds/ Compound Library	Hit Rate ^a^	Binding Affinity of Hits	Literature
**Structure-based virtual screening**					
Docking	μ-opioid receptor	3 M/ZINC	23/23	2.3–14 μM	Manglik et al., 2016 [16]
Docking	Mas-related G protein- coupled receptor X2 (MRGPRX2)	3.7 M/ZINC	20/20	<10 μM	Lansu et al., 2017 [17]
Docking	Histamine H1 receptor	100 K/ZINC	19/26 (73%)	6 nM–10 μM	De Graaf et al., 2011 [18]
Docking	Histamine H4 receptor	8.7 M/ZINC	16/255 (6%)	85–1480 nM	Kiss et al., 2008 [19]
Docking	Histamine H4 receptor	7 K/Bioprojet chemical library	28/120 (23%)	4 nM–16 μM	Levoin et al., 2017 [20]
Docking	Melanin-concentrating hormone receptor 1 (MCH-R1)	187 K/In-house collection [21]	6/129 (5%)	7–20 μM	Cavasotto et al., 2008 [22]
Docking	Chemokine receptor CCR5	1.6 M/8 vendors	10/59 (17%)	5–200 μM	Kellenberger et al., 2007 [23]
Docking	Adenosine receptor A2A	1.4 M/ZINC	7/20 (35%)	200 nM–9 μM	Carlsson et al., 2010 [24]
Docking	Adenosine receptor A2A	4.3 M/Molsoft ScreenPub	23/56 (41%)	<10 μM	Katritch et al., 2010 [25]
Docking	β2-adrenergic receptor	1 M/ZINC	6/25 (24%)	<4 μM	Kolb et al., 2009 [26]
Docking	Dopamine D2 receptor	6.5 M/Enamine	10/21 (48%)	58 nM–25 μM	Kaczor et al., 2016 [27]
Docking	Choline acetyltransferase (ChAT)	300 K/Asinex Gold and Platinum collection library	3/35 (9%)	7–26 μM	Kumar et al., 2017 [28]
Docking	Tau fibrils	62 K/FDA-approved small molecule drugs and ChemBridge CNS-set	4/46 (9%)	<5 μM	Seidler et al., 2022 [29]
Docking	Dopamine D3 receptor	1.5 M/ChemDiv	27/37(73%)	<10 μM	Jin et al., 2023 [30]
Pharmacophore	Formylpeptide receptor (FPR)	480 K/Chemical Diversity Laboratories [31]	30/4324 (0.7%)	1–32 μM	Edwards et al., 2005 [32]
Pharmacophore	complement component 3a receptor 1 (C3AR1)	-/In-house collection	4/157 (3%)	<10 μM	Klabunde et al., 2009 [33]
Pharmacophore	Alpha-synuclein fibrils	10 M/ZINC15	2/17 (12%)	10–490 nM	Ferrie et al., 2020 [2]
Pharmacophore	Histamine H4 receptor	22 M/ZINC12	3/291 (1%)	<10 μM	Ko et al., 2018 [34]
Pharmacophore Docking	Sphingosine kinase 1 (SphK1)	147/Custom-selected Library	3/16 (19%)	12–60 μM	Vettorazzi et al., 2017 [35]
Pharmacophore Docking	Serotonin transporter (SERT)	1 M/ZINC	2/15 (13%)	17–38 μM	Manepalli et al., 2011 [36]
Pharmacophore Docking	Thyrotropin-releasing hormone receptor1 (TRH-R1)	1 M/ZINC	100/100	Sub μM–μM	Engel et al., 2008 [37]
Pharmacophore Docking	Alpha1A adrenergic receptor	23 K/MDL Drug Data Report	37/80 (46%)	<10 μM	Evers et al., 2005 [38]
Pharmacophore Docking	Neurokinin-1 (NK1) receptor	827 K/7 databases	1/7 (14%)	0.25 μM	Evers et al., 2004 [39]
Machine learning	Acetylcholinesterase (AchE)	15 M/Enamine REAL database	10/23(43%)	<50 μM	Adeshina et al., 2020 [40]
**Ligand-based virtual screening**					
Pharmacophore	Metabotropic glutamate receptor 5 (mGluR5)	194 K/Asinex Gold compound collection	9/27 (33%)	<70 μM	Renner et al., 2005 [41]
Pharmacophore	Metabotropic glutamate receptor 1 (mGluR1)	201 K/Asinex Gold Collection	6/23 (26%)	0.75–>40 μM	Noeske et al., 2007 [42]
2D-QSAR	Sigma 2 receptor	2 K/DrugBank	10/34 (29%)	140 nM–μM	Yu et. al., 2021 [43]
2D Fingerprint	Sigma 2 receptor	47 M/MCule Inc.	12/46 (26%)	0.6–700 nM	Kim et al., 2022 [3]
**Ligand- and structure-based virtual screening**					
2D/3D-QSAR Docking	Sigma 2 receptor	1517/Seaweed Metabolite and ChEBI	15/15	0.6–5.3 nM	Floresta et al., 2018 [44]
2D Fingerprint Pharmacophore	Melanin-concentrating hormone 1 receptor (MCH-1)	615 K/24 Vendors	15/795 (1.9%)	1–30 μM	Clark et al., 2004 [21]
Similarity Pharmacophore Docking	Free fatty acid receptor 1 (FFAR1)	2.6 M/ZINC	6/52 (12%)	<10 μM	Tikhonova et al., 2008 [45]
Pharmacophore Docking	Subtype six serotonin receptor (5-HT6)	-/Princeton BM and ChemBridge	14/92 (15%)	<1 μM	Staron et al., 2020 [46]
Pharmacophore Docking	5-HT7 receptor (5-HT7R)	730 K/Enamine Screening Collection	2/26 (8%)	197–265 nM	Kurczab et al., 2010 [47]

^a^ Hit rate was calculated from the number of compounds that have been measured binding affinity to the number of compounds submitted to in vitro binding assay from virtual hits.

## Data Availability

Data is contained within the article and Supplementary Material.

## Supplementary Materials

The following supporting information can be downloaded at: https://www.mdpi.com/article/10.3390/ph16020317/s1, Table S1: The chemical properties and bio-behavior predictions of compounds used in the BOILED-Egg plot.
